# Altered vaspin levels in hypothyroid patients: A potential marker of metabolic dysregulation

**DOI:** 10.1097/MD.0000000000047620

**Published:** 2026-02-06

**Authors:** Gökhan Köker, Kübra Çerçi, Muhammed Ali Coşkuner, Lütfullah Zahit Koç, Hamit Yaşar Ellidağ, Jehat Kiliç, Bilgin Bahadir Başgöz

**Affiliations:** a Department of Internal Medicine, University of Health Sciences, Antalya Training and Research Hospital, Antalya, Turkey; b Department of Internal Medicine, University of Health Sciences, Antalya State Hospital, Antalya, Turkey; c Department of Biochemistry, University of Health Sciences, Antalya Training and Research Hospital, Antalya, Turkey; d Department of Internal Medicine, University of Health Sciences, Gazi Yaşargil Training and Research Hospital, Diyarbakir, Turkey.

**Keywords:** adipokines, hypothyroidism, metabolic dysregulation, vaspin

## Abstract

This study investigates alterations in vaspin levels in hypothyroid patients and their role in metabolic processes and thyroid function. By analyzing the correlation between vaspin levels and hypothyroidism, the research aims to elucidate metabolic dysregulation in thyroid disorders and the potential link between thyroid hormones and adipose tissue function. This study included patients with hypothyroidism and healthy controls. Comprehensive data and laboratory findings were collected from hospital electronic health records. Vaspin levels were analyzed to explore the association with hypothyroidism. The Homeostasis Model Assessment of Insulin Resistance (HOMA-IR) was also calculated. The study included 90 participants: 54 in the hypothyroid group and 36 in the control group. The hypothyroid group had a significantly higher age (49.04 ± 11.89 vs 43.53 ± 9.76 years, *P* = .019). Thyroid function tests showed substantially higher thyroid-stimulating hormone and lower fT4 levels in the hypothyroid group, confirming impaired thyroid function. Notably, vaspin levels were significantly lower in the hypothyroid group than in the control group (2.24 ng/mL vs 3.51 ng/mL, *P* = .008). This finding suggests that hypothyroidism may lead to altered adipokine regulation, potentially contributing to metabolic dysfunction. No significant differences were observed between the groups for lipid profile, glucose metabolism, insulin resistance (HOMA-IR), hematological, or liver enzymes. This study found lower vaspin levels in hypothyroid patients, which may be related to metabolic dysregulation. Vaspin could serve as a therapeutic target for addressing metabolic disturbances in hypothyroidism. Further research is needed to explore its clinical implications.

## 1. Introduction

Adipose tissue functions not only as an energy reservoir but also as an active endocrine organ that secretes a variety of bioactive molecules collectively known as adipokines. These mediators – including adiponectin, leptin, resistin, and vaspin – play crucial roles in glucose and lipid metabolism, insulin sensitivity, and inflammatory regulation. Dysregulation of adipokine secretion has been implicated in several metabolic and endocrine disorders such as obesity, metabolic syndrome, and diabetes.^[1,2]^

Thyroid dysfunction, particularly hypothyroidism, profoundly affects basal metabolism and body composition. Thyroid hormones modulate adipokine synthesis and secretion, suggesting a bidirectional relationship between thyroid activity and adipose tissue homeostasis. Clinical and experimental studies indicate that adipokines may partially mediate the metabolic effects of thyroid hormones and contribute to components of metabolic syndrome.^[3,4]^ Beyond metabolic regulation, thyroid hormones exert widespread systemic effects, including on the central nervous system, where subtle thyroid dysfunction may lead to neurocognitive and neuropsychiatric manifestations even in the absence of overt hormonal imbalance. These findings underscore the complex multisystem impact of thyroid disorders and their potential interaction with adipose tissue-derived signaling molecules.^[5]^ In this context, vaspin has emerged as a promising adipokine that may reflect metabolic alterations associated with thyroid disorders.^[6]^

Vaspin, a member of the serpin (serine protease inhibitor) family, is primarily secreted from visceral adipose tissue and has been identified as a regulator of insulin sensitivity and glucose metabolism. Experimental studies have suggested that vaspin may exert anti-inflammatory and insulin-sensitizing effects through modulation of protease activity, particularly kallikrein-related enzymes.^[7,8]^ Owing to its metabolic role, vaspin has been associated with various endocrine and metabolic disorders, including obesity, type 2 diabetes mellitus, and polycystic ovary syndrome. Emerging evidence further suggests that thyroid hormones may influence vaspin expression through alterations in adipocyte function, systemic inflammation, and metabolic homeostasis, supporting a potential mechanistic link between thyroid dysfunction and circulating vaspin levels.^[9]^ However, findings remain inconsistent, indicating that the regulatory mechanisms are still not fully understood.^[7,8]^

Previous studies have reported inconsistent findings regarding the relationship between thyroid dysfunction and circulating vaspin levels. Some authors have found elevated vaspin concentrations in patients with hypothyroidism, whereas others observed decreased or unchanged levels.^[10,11]^ These discrepancies suggest that vaspin regulation may depend on disease severity, body composition, or metabolic status, indicating a complex interaction between thyroid function and adipose tissue signaling.

Based on these observations, the present study aimed to investigate alterations in serum vaspin levels among hypothyroid patients compared with euthyroid controls and to explore their potential association with metabolic disturbances.

## 2. Materials and methods

This study was designed as a prospective case–control study and included patients monitored for thyroid dysfunction at the University of Health Sciences Antalya Training and Research Hospital between May 2024 and July 2024. The control group consisted of individuals with normal thyroid function and had no evidence of endocrine or metabolic disease. All biochemical and clinical data, including serum vaspin measurements, were collected prospectively under standardized study conditions.

Patients with overt hypothyroidism were included, defined as serum thyroid-stimulating hormone (TSH) levels >4.5 mIU/L accompanied by free thyroxine (fT4) levels lower than 0.8 ng/dL, according to institutional laboratory reference intervals. All hypothyroid patients included in this study had autoimmune thyroiditis, confirmed by elevated thyroid autoantibodies (anti-thyroid peroxidase antibodies [anti-TPO], and anti-thyroglobulin [anti-TG] positivity). The selection was intentional to ensure a homogeneous study population with autoimmune etiology of hypothyroidism.

Individuals with subclinical hypothyroidism (defined as normal fT4 levels with elevated TSH) were not included, and all participants were newly diagnosed and untreated at the time of enrollment. Patients with diabetes mellitus, chronic liver or kidney disease, cardiovascular disease, active infection, malignancy, pregnancy, alcohol use, or smoking were excluded. Importantly, individuals who were using medications known to affect lipid metabolism, glucose homeostasis, body weight, or adipokine levels were excluded from the study. These medications included lipid-lowering agents (e.g., statins or fibrates), antidiabetic drugs, systemic corticosteroids, and weight-loss therapies such as glucagon-like peptide-1 receptor agonists (e.g., liraglutide, semaglutide) and orlistat.

The control group consisted of age- and sex-comparable euthyroid individuals (TSH 0.45–4.5 mIU/L; fT4 0.8–1.8 ng/dL) with no known endocrine or metabolic disorders.

Demographic information, including age and gender, was systematically documented. Additionally, we gathered a complete set of blood parameters, which included hemoglobin (Hb), hematocrit (Htc), mean corpuscular volume (MCV), white blood cell count (WBC), neutrophils, lymphocytes, monocytes, and platelet levels. Biochemical markers including glucose, creatinine, aspartate aminotransferase (AST), alanine aminotransferase (ALT), total cholesterol, high-density lipoprotein (HDL), low-density lipoprotein (LDL), triglycerides (TG), and insulin were also measured.

Furthermore, an in-depth assessment of thyroid function was conducted, including measurements of free triiodothyronine (fT3), fT4, TSH, anti-TPO and anti-TG. As part of our investigation, vaspin levels were also analyzed to explore potential correlations. Serum vaspin levels were measured using the ETI-MAX 3000 analyzer (DiaSorin, Saluggia, Italy) with the enzyme-linked immunosorbent assay (ELISA) technique.

The Homeostasis Model Assessment of Insulin Resistance (HOMA-IR) is a valuable method for evaluating insulin resistance, which plays a critical role in various metabolic conditions. The following formula is utilized to obtain the HOMA-IR. HOMA-IR = [glucose concentration (in nmol/L) × insulin concentration (in µU/mL),/22.5]. This calculation explicitly requires measuring of fasting glucose and insulin levels, as they provide a clearer picture of how the body’s insulin response is functioning during a resting state. The resulting HOMA-IR allows healthcare professionals to estimate the degree of insulin resistance a person may be experiencing. Generally, elevated HOMA-IR indicate increased insulin resistance, which is often linked to health issues such as overweight, obesity, and type 2 diabetes. By analyzing these values, clinicians can formulate personalized strategies for managing and improving an individual’s metabolic health.^[12]^

The study adhered strictly to the Helsinki Declaration, ensuring that ethical standards were maintained throughout the research process. Before commencing the study, we obtained approval from our hospital’s ethics committee on April 04, 2024 with the reference number 4/10, validating the ethical considerations taken in our research approach.

The statistical analyses were conducted using the Statistical Package for Social Sciences (Chicago) version 26.0. The sample size was determined based on an a priori power analysis to ensure sufficient statistical power for detecting a statistically significant difference in vaspin levels between hypothyroid patients and controls. This approach allowed us to calculate the necessary number of participants in each group while considering practical feasibility constraints. The normality of the data was assessed using the Shapiro–Wilk test. Data that followed a normal distribution were expressed as mean ± standard deviation, while skewed data were presented as median (interquartile range). For continuous variables that adhered to a normal distribution, comparisons were made using the Student *t* test. The Mann–Whitney U test was used when the data did not follow a normal distribution. Categorical variables were analyzed using the Chi-square test. A *P*-value of <.05 was considered statistically significant.

## 3. Results

The study included 90 participants: 54 in the hypothyroid group and 36 in the control group. While the mean age of participants in the hypothyroid group was significantly higher than that of the control group (*P* = .019), the frequency of females was similar (*P* = .406) (Table 1).

**Table 1:** The comparison of laboratory findings and vaspin levels among participants.

	Total (n = 90)	Hypothyroid (n = 54)	Control (n = 36)	*P*-values
Gender				
Female, n (%)	76 (84.4)	47 (52.2)	29 (32.2)	.406
Male, n (%)	14 (15.6)	7 (7.8)	7 (7.8)	
Age, mean (SD)	46.83 (11.36)	49.04 (11.89)	43.53 (9.76)	**.019**
Hb, mean (SD)	12.93 (1.58)	12.74 (1.75)	13.21 (1.23)	.137
Htc, median (IQR)	38.75 (5.00)	39.30 (4.70)	39.35 (5.23)	.542
MCV, median (IQR)	82.25 (7.57)	86.50 (11.95)	84.70 (6.10)	.281
WBC, median (IQR)	7.10 (2.78)	7.00 (2.90)	7.05 (2.33)	.539
Neutrophile, median (IQR)	4.20 (1.73)	4.10 (1.95)	4.10 (1.60)	.537
Lymphocyte, median (IQR)	2.25 (0.93)	2.20 (1.00)	2.25 (0.95)	.683
Monocyte, median (IQR)	0.50 (0.20)	0.50 (0.20)	0.50 (0.20)	.630
Platelet, mean (SD)	274.53 (62.73)	275.04 (69.58)	273.78 (51.73)	.922
Glucose, median (IQR)	90.00 (15.25)	88.00 (13.00)	94.00 (17.50)	.201
Insulin, median (IQR)	6.78 (6.32)	6.86 (7.58)	6.47 (3.46)	.980
HOMA-IR, median (IQR)	1.49 (1.57)	1.47 (1.73)	1.45 (0.82)	.958
Creatinine, median (IQR)	0.81 (0.17)	0.78 (0.15)	0.83 (0.18)	.287
AST, median (IQR)	18.50 (6.00)	19.00 (7.00)	17.50 (5.00)	.396
ALT, median (IQR)	17.00 (12.00)	16.00 (8.00)	15.50 (10.00)	.315
Total cholesterol, median (IQR)	187 (65)	188 (60)	186 (64)	.724
HDL, median (IQR)	52.00 (15.50)	50.00 (19.50)	51.50 (15.75)	.993
LDL, median (IQR)	117.00 (50.00)	123.00 (59.50)	117.00 (51.50)	.676
TG, median (IQR)	104.00 (92.00)	97.00 (67.50)	113.50 (117.50)	.597
TSH, median (IQR)	6.88 (7.84)	9.97 (14.31)	2.15 (1.71)	**<.001**
fT4, mean (SD)	0.70 (0.15)	0.64 (0.14)	0.78 (0.13)	**<.001**
fT3, mean (SD)	3.33 (0.44)	3.27 (0.49)	3.42 (0.34)	.085
Anti-TG, median (IQR)	0.90 (39.46)	10.00 (621.20)	0.30 (0.80)	**<.001**
Anti-TPO, median (IQR)	13.80 (363.40)	358.80 (774.75)	0.50 (1.08)	**<.001**
Vaspin, median (IQR)	2.26 (1.81)	2.24 (1.34)	3.51 (4.14)	**.008**

Bold values indicate statistically significant differences (*P* < .05).

ALT = alanine aminotransferase, Anti-TG = anti-thyroglobulin, Anti-TPO = anti-thyroid peroxidase antibodies, AST = aspartate aminotransferase, fT3 = free triiodothyronine, fT4 = free thyroxine, Hb = Hemoglobin, HDL = high-density lipoprotein, HOMA-IR = homeostatic model assessment of insulin resistance, Htc = hematocrit, IQR = interquartile range, LDL = low-density lipoprotein, MCV = mean corpusculer volume, SD = standard deviation, TG = triglycerides, TSH = thyroid-stimulating hormone, WBC = white blood cell.

### 3.1. Thyroid function and autoimmune markers

The hypothyroid group showed significantly higher TSH and lower fT4 levels than the control group (*P* <.001 for both), indicating impaired thyroid function Although fT3 levels were lower in the hypothyroid group, the difference was not reached significant level (*P* = .085). Additionally, both anti-TG and anti-TPO levels were markedly elevated in the hypothyroid group (*P* <.001 for both), suggesting an autoimmune basis for hypothyroidism in these patients (Table 1).

### 3.2. Metabolic and biochemical parameters

Lipid profile parameters, including total cholesterol, HDL, LDL and TG, were compared between groups, and all total cholesterol, HDL, LDL or TG were found to be similar (*P* >.05 for all) (Table 1). Similarly, glucose, insulin, and HOMA-IR levels did not differ significantly between the 2 groups (*P* >.05 for all) (Table 1). These results suggest that thyroid dysfunction did not considerably impact glucose metabolism or insulin resistance in this cohort.

### 3.3. Hematological and liver function markers

No significant differences were observed between the groups regarding hematological parameters, including Hb, Htc levels, and platelet counts (*P* >.05 for all). Also, WBC counts and the distribution of neutrophils, lymphocytes, and monocytes showed no significant differences. Similarly, the hypothyroid and control groups were compared in terms of AST and ALT enzyme levels, and the results revealed no difference (*P* >.05 for both) (Table 1).

### 3.4. Vaspin levels

One of the most notable findings was the significant difference in vaspin levels between the 2 groups. The hypothyroid group had significantly lower vaspin levels than the control group (2.24 ng/mL vs 3.51 ng/mL, *P* = .008). Vaspin, an adipokine associated with insulin resistance and obesity, may indicate altered adipokine regulation in hypothyroid patients, which could have implications for metabolic dysfunction (Table 1).

## 4. Discussion

In this comprehensive study, we focused on a cohort of 54 patients diagnosed with hypothyroidism who were closely monitored at our hospital. We also included a control group of 36 healthy individuals to provide a robust comparison. The primary objective of this research was to investigate potential alterations in the levels of vaspin, a protein primarily secreted by adipose tissue, in hypothyroid patients. By analyzing the differences in vaspin release between these 2 groups, we aimed to deepen our understanding of the metabolic changes associated with hypothyroidism and its impact on adipose-derived factors.

The intricate relationship between hypothyroidism and obesity has been a subject of significant research, revealing noteworthy connections between TSH levels and various adipocytokines – hormones secreted by adipose tissue. A study conducted by Çinar et al specifically examined hypothyroid patients, categorizing them into 3 distinct groups: those with overt hypothyroidism, those with subclinical hypothyroidism, and individuals with normal thyroid function, the findings indicated that vaspin levels were markedly elevated in the hypothyroid group, though this increase was statistically insignificant, suggesting a potential link between thyroid hormone levels and adipose tissue signaling.^[10]^ In another study, vaspin levels were statistically significantly higher in the hypothyroid group.^[11]^ Similarly, studies on rats have shown that vaspin levels are positively correlated with TSH and are elevated in hypothyroid rats.^[13,14]^ In contrast, our research yielded different results. We found that vaspin levels were significantly lower in patients with hypothyroidism, with an average of 2.24 ng/mL. In comparison, the control group, consisting of individuals with normal thyroid function, exhibited average vaspin levels of 3.51 ng/mL. The difference in vaspin levels between the 2 groups was notable and statistically significant (*P* = .008), indicating a compelling divergence in the relationship between vaspin levels and thyroid function in our study population. This contrast opens up intriguing avenues for further investigation into the role of vaspin in the context of thyroid dysfunction and its potential implications for weight management and metabolic health.

Hypothyroidism can change the complete blood count, with MCV and mean corpuscular hemoglobin (MCH) being the most significant parameters affected. Studies have shown a decrease in MCV and MCH in hypothyroidism.^[13]^ In our research, the median MCV value for hypothyroid patients was 86.50 (11.95). In comparison, the control group had an median MCV value of 84.70 (6.10). Although the MCV was higher in the hypothyroid group, this difference was insignificant (*P* = .281). Additionally, the changes in WBC counts, as well as neutrophil, lymphocyte, monocyte, and platelet values, were not significantly different from those in the control group, with *P*-values of .539, .537, .683, .630, and .922, respectively. This similarity might be associated with the limited size of our patient population.

Hypothyroidism can cause liver damage, but this damage is typically mild and reversible. A study conducted by Arora et al on hypothyroid patients found that levels of ALT, AST and alkaline phosphatase were elevated, but these levels decreased after treatment.^[14]^ However, no significant differences were observed in these values compared to the control group. Our study also found higher AST and ALT values compared to the control group, but this difference was not statistically significant (*P* = .396, *P* = .315).

Additionally, total cholesterol, TG and LDL levels tend to increase in patients with hypothyroidism. In the study conducted by Teixeira et al, these lipid profiles were remarkably worse in hypothyroid patients.^[15]^ Our study observed that total cholesterol and LDL levels were higher, while TG levels were lower than in the control group. However, these differences were not statistically significant, with *P*-values of .724, .676, and .597, respectively.

Insulin resistance and hypothyroidism are 2 extensively researched topics, with high HOMA-IR commonly observed in patients with hypothyroidism. A study conducted by Vyakaranam et al found that insulin levels and HOMA-IR scores were elevated in patients with subclinical hypothyroidism, and these findings were statistically significant.^[16]^ In our study, the insulin and HOMA-IR scores in hypothyroid patients were measured at 6.86 (±7.58) and 1.47 (±1.73), respectively. These values were higher than those in the control group, which had insulin levels 6.47 (±3.46) and HOMA-IR of 1.45 (±0.82). However, these differences did not reach a significant level (*P*-values: .980 and .958, respectively).

The primary limitations of this study are its single-center design and the relatively small sample size of patients included in the analysis. While this study provides valuable insights into vaspin levels in hypothyroid patients, its external validity is limited due to factors such as potential selection bias, small sample size, and lack of multi-center validation. Therefore, to enhance generalizability, future studies should include larger, multi-center cohorts, diverse ethnic groups, and evaluate comorbidities, thyroid treatment, and longitudinal changes in vaspin levels.

## 5. Conclusion

In conclusion, the study underscores significant differences in thyroid function and autoimmune markers between hypothyroid patients and controls. Hypothyroid patients exhibited higher TSH and lower fT4 levels, along with elevated anti-TG and anti-TPO antibody levels, indicating autoimmune thyroid disease. While no significant differences were observed in lipid profile, glucose metabolism, or hematological and liver function markers, the significantly lower vaspin levels in hypothyroid patients suggest a potential metabolic disturbance associated with thyroid dysfunction. These findings may contribute to a deeper understanding of the metabolic consequences of hypothyroidism and offer insight into potential therapeutic targets.

## Author contributions

**Conceptualization:** Kübra Çerçi, Muhammed Ali Coşkuner.

**Data curation:** Gökhan Köker, Kübra Çerçi, Hamit Yaşar Ellidağ.

**Formal analysis:** Kübra Çerçi, Hamit Yaşar Ellidağ, Bilgin Bahadir Başgöz.

**Investigation:** Gökhan Köker.

**Methodology:** Gökhan Köker, Kübra Çerçi, Muhammed Ali Coşkuner.

**Project administration:** Muhammed Ali Coşkuner, Lütfullah Zahit Koç, Jehat Kiliç.

**Resources:** Lütfullah Zahit Koç.

**Software:** Gökhan Köker, Muhammed Ali Coşkuner, Lütfullah Zahit Koç.

**Supervision:** Muhammed Ali Coşkuner.

**Validation:** Lütfullah Zahit Koç, Bilgin Bahadir Başgöz.

**Writing – original draft:** Gökhan Köker, Jehat Kiliç.

**Writing – review & editing:** Muhammed Ali Coşkuner, Bilgin Bahadir Başgöz.
